# A complicated case of whole-lung lavage: a case report

**DOI:** 10.3389/fmed.2023.1225167

**Published:** 2023-07-19

**Authors:** Simone Petrarulo, Manuel Lucchin, Stefano Oldani, Alessandra Dubini, Sara Piciucchi, Alberto Gori, Luca Aiello, Stefano Maitan, Paolo Spagnolo, Claudia Ravaglia, Venerino Poletti

**Affiliations:** ^1^Respiratory Disease Unit, Department of Cardiac Thoracic, Vascular Sciences and Public Health, University of Padova, Padova, Italy; ^2^Respiratory and Critical Care Unit, Department of Clinical Integrated and Experimental Medicine (DIMES), IRCCS Azienda Ospedaliero Universitaria Bologna, University Hospital Sant'Orsola—Malpighi, Bologna, Italy; ^3^Department of Medical Specialities, Pulmonology Unit, GB Morgagni—L. Pierantoni Hospital, Forlì, Italy; ^4^Department of Pathology, GB Morgagni—L. Pierantoni Hospital, Forlì, Italy; ^5^Department of Radiology, GB Morgagni—L. Pierantoni Hospital, Forlì, Italy; ^6^Section of Anesthesia and Intensive Care, Department of Surgery, GB Morgagni—L. Pierantoni Hospital, Forlì, Italy; ^7^Department of Respiratory Diseases and Allergy, Aarhus University Hospital, Aarhus, Denmark; ^8^Department of Medical and Surgical Sciences (DIMEC), University of Bologna/Forlì Campus, Forlì, Italy

**Keywords:** whole-lung lavage, pulmonary alveolar proteinosis, lysinuric protein intolerance, case report, pneumothorax

## Abstract

**Introduction:**

We report a life-threatening case of severe respiratory failure due to a pulmonary alveolar proteinosis (PAP) secondary to lysinuric protein intolerance (LPI), complicated by a pre-existing right pneumothorax, which we treated using a rescue whole-lung lavage (WLL). To date, in the literature, there are no cases of WLL performed in this condition.

**Clinical condition:**

Patient was referred to our center because of rapidly worsening dyspnea and deterioration of gas exchange, caused by a secondary form of PAP which required an immediate therapeutic option such as the one offered by WLL. On physical examination, bilateral crackles were present, and peripheral blood oxygen saturation was 78% on oxygen with a FiO_2_ of 40%.

**Interventions:**

After stabilizing the clinical conditions with oxygen therapy erogated through a high-flow nasal cannula, shortly after admission, we performed a rescue WLL among two procedures. The procedure was very effective, and the patient was later discharged without oxygen therapy and in good clinical condition.

**Conclusion:**

Our case report represents a chance to help fill the gap of knowledge relative to secondary forms of PAP. The patient we presented suffers from a very rare genetic condition (LPI) that only has a few reported cases in the literature and has a very low prevalence which makes it difficult to produce the affected people:newborns ratio. We believe that difficult and rare cases like this one can improve our understanding of the disease and, most importantly, of how much the only therapeutic option we had, a rescue WLL, is effective to improve gas exchange and radiological features, despite being performed in these severe respiratory conditions.

## Introduction

A 32-year-old lifelong non-smoker woman with a life-threatening respiratory failure due to a PAP secondary to LPI, complicated by a pre-existing right pneumothorax, was referred to us to perform a rescue whole-lung lavage procedure leading to improve clinical and functional conditions. She never performed a WLL previously. WLL is a technique that was developed in the 1960's with the purpose of removing lipoproteinaceous material that accumulates in the alveoli and it is the most commonly accepted therapy for patients with symptomatic PAP. Despite the right pneumothorax and the severe respiratory failure, the procedure was conducted in general anesthesia, and a WLL, performed with a chest tube inserted, was completed in two phases and it has proven effective to improve gas exchange and radiological features.

## Case presentation

A 32-year-old lifelong non-smoker Caucasian woman was referred to the G.B. Morgagni—L. Pierantoni Hospital in April 2023 due to a severe respiratory failure caused by PAP syndrome complicated by a right pneumothorax.

Since an early age, the patient has been followed by the pediatric department of her hometown hospital, where in 2002 she received the diagnosis of lysinuric protein intolerance. The diagnosis was definite because of the patient's clinical and biochemical situation, as well as molecular analysis, which revealed the presence of mutations in the SLC7A7 gene. The patient also has a homozygous mutation of the MTHFR gene, which codes for methylenetetrahydrofolate reductase, resulting in high homocysteine levels which lead to blood vessels damage and finally strongly increase the risk of blood clots.

Her past medical history was also remarkable for a Fanconi-like tubulopathy, low stature secondary due to GH deficit, hyperlipidemia, osteoporosis, and gastritis; from 2010, the patient suffered a progressive accumulation of lipoproteinaceous material in the lungs, resulting in dry cough and slight exertional dyspnea, with diffuse ground glass opacities (GGOs) on HRCT scans, which remained stable during radiological follow-up. On the last follow-up visit, only 1 month previously, the last pulmonary function tests (PFTs) revealed a restrictive respiratory disorder (forced vital capacity—FVC: 57% predicted; forced expiratory volume in the first second—FEV1: 55% predicted; Tiffeneau Index 89% predicted; the patient was not able to perform diffusion capacity for carbon monoxide—DLCO).

The patient was referred to the G.B. Morgagni—L. Pierantoni Hospital in April 2023 due to a rapid onset of acute respiratory failure, complicated by a right pneumothorax. Two weeks before our admission, the patient experienced a respiratory tract infection treated with broad-spectrum antibiotics and chest tube insertion.

**Figure 1:**
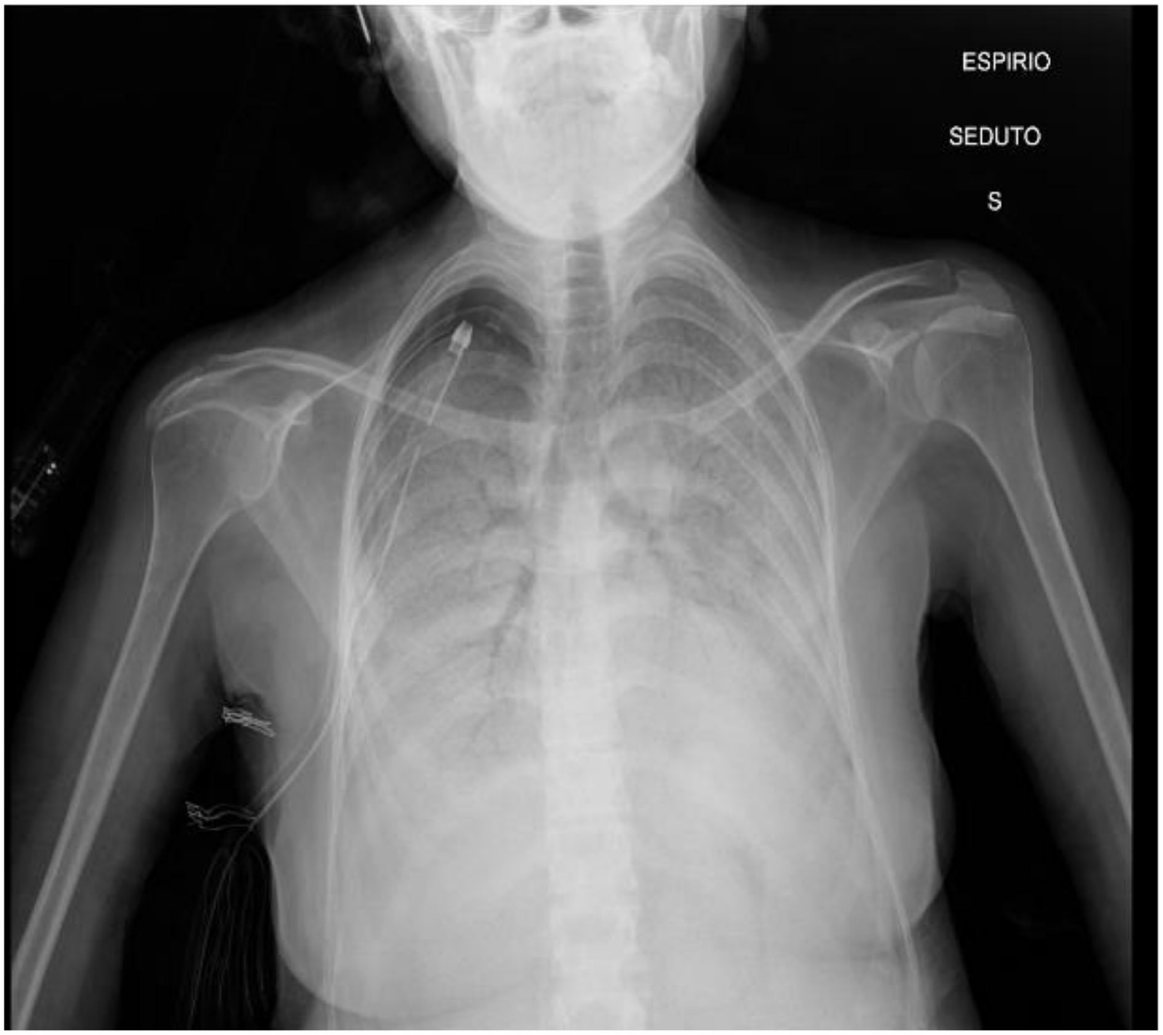
Chest X-ray on admission.

On admission, physical examination revealed a blood pressure of 120/75 mmHg, a heart rate of 85 bpm, a respiratory rate of 26 breaths/min, and a hemoglobin oxygen saturation of 78% on oxygen (FiO_2_ 40%) delivered through the nasal cannula; she was afebrile. Subsequently, arterial blood gas (ABG) analysis showed a severe acute type 1 respiratory failure (pH 7.37, pO_2_ 34.5 mmHg, pCO_2_ 40.9 mmHg, PaO_2_/FiO_2_ 85). The patient was quickly adapted to a high-flow nasal cannula (HFNC) with a FiO_2_ of 55% and a flow of 60 L/min, which rapidly improved gas exchange and increased hemoglobin oxygen saturation to 91%. Chest auscultation revealed severely reduced respiratory sound and bilateral crackles, while cardiac auscultation was normal. Chest x-ray (Figure 1) showed a right apical pneumothorax (with chest tube previously inserted) and extensive diffuse bilateral consolidations with bronchus sign.

Subsequently, high-resolution computed tomography (HRCT) of the chest (**Figure 3A**) showed areas of diffuse ground glass with superimposed interlobular septal thickening (“crazy paving” pattern) and a right pneumothorax.

Blood tests showed increased neutrophil count (WBC 16.7 × 10^9^/L, neutrophils 14.9 × 10^9^/L) and reduced hemoglobin levels (Hb 8.0 g/dl). LDH was markedly elevated (1,404 U/L), while CRP and procalcitonin were within normal range.

Therefore, the decision was made to proceed with WLL, which was performed on two different days. Under general anesthesia, airway management and lung isolation were achieved using a double-lumen endotracheal tube (DL-ETT). Bronchoscopy confirmed the correct placement of the DL-ETT; a bronchoalveolar lavage revealed a foamy, thick, whitish material, which on rapid on-site evaluation (ROSE) was shown to contain lymphocytes and acellular proteinaceous eosinophilic material.

Afterward, the patient was positioned on left lateral decubitus, and single lung ventilation was set with FiO_2_ 100%. During the first procedure, a total of 5,500 mL, in aliquots of 1,000 mL, of warm (37°C) sterile saline solution (0.9% NaCl) were instilled in the right lung (**Figure 3B**). At each cycle, physiotherapists performed chest percussion maneuvers to optimize proteinaceous fluid clearance over a period of 2 min when the maximum volume has been infused and during the drainage phase.

Fluid input and output were carefully monitored and registered. However, for safety reasons, the procedure was prematurely discontinued because the patient showed a SpO_2_ of 42% during the fifth aliquots infusion, probably due to a rapid worsening of mismatch in ventilation/perfusion ratio (V'A/Q') during the filling phase.

**Figure 2:**
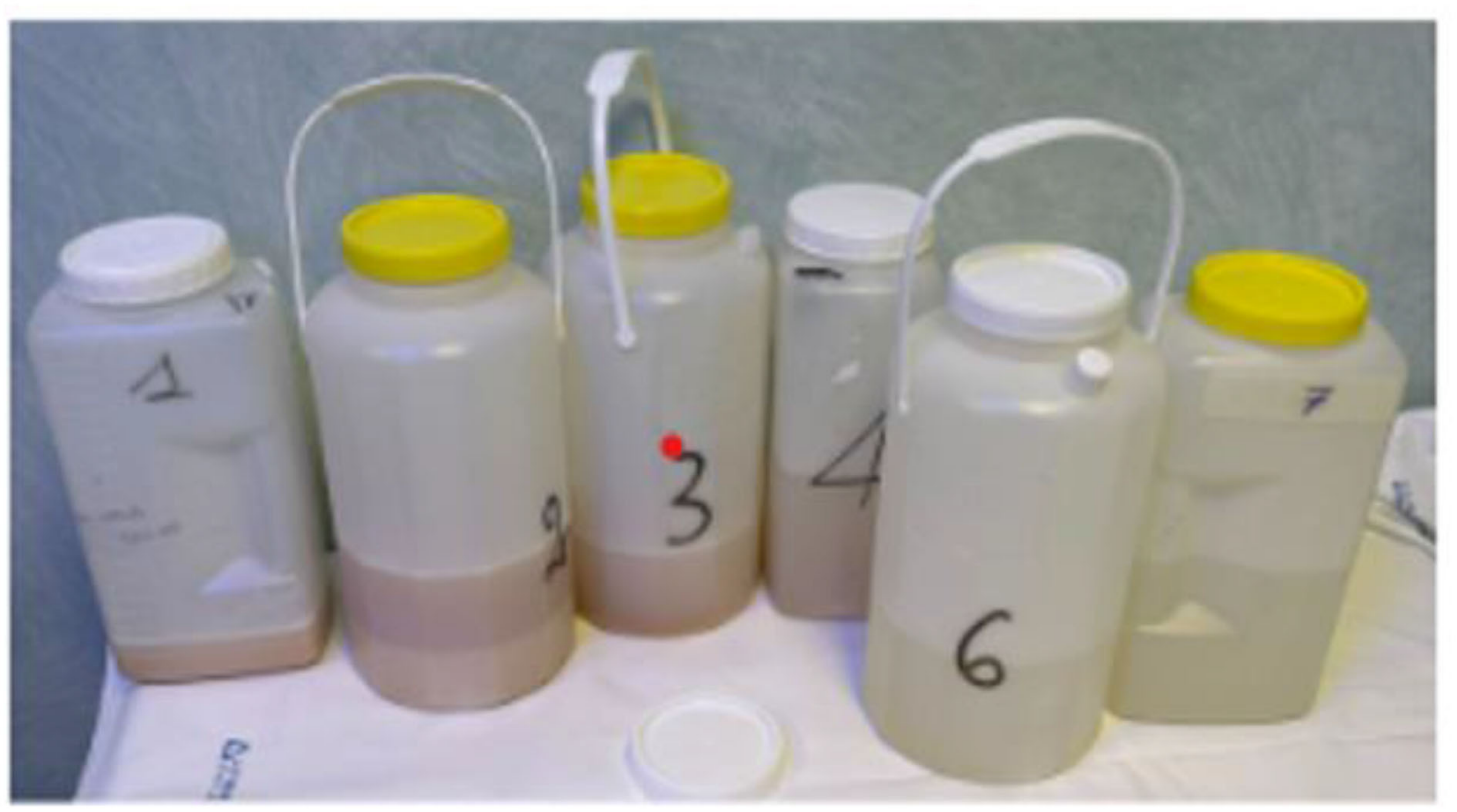
Fluids from WLL.

**Figure 3:**
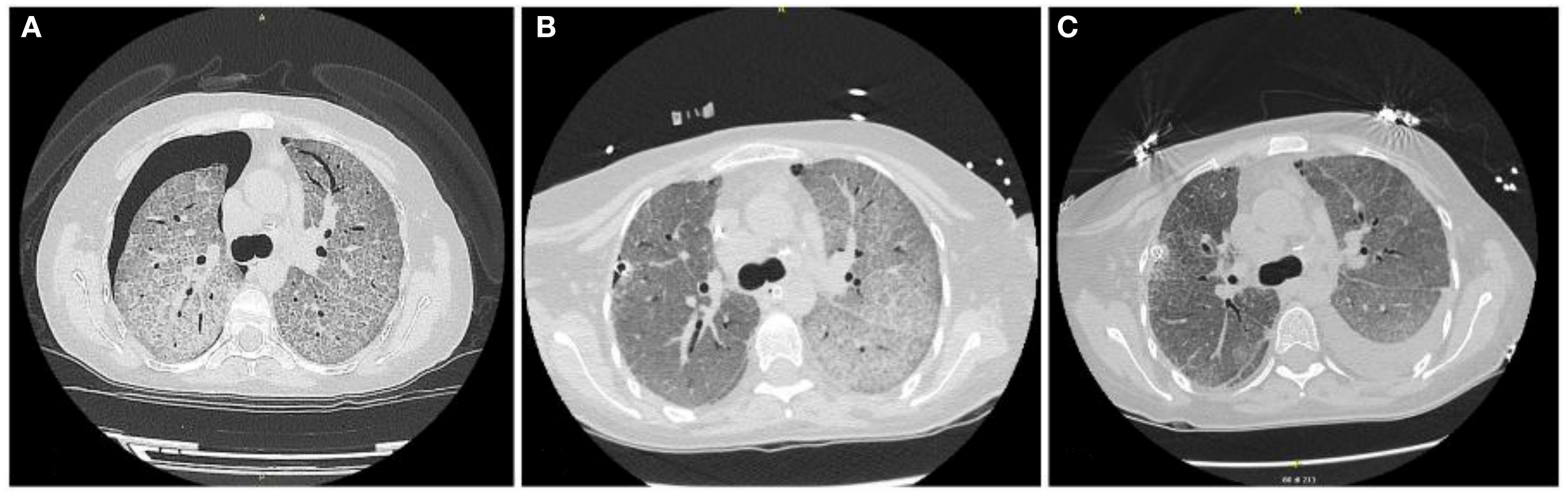
**(A)** HRTC scan at admittance; **(B)** HRTC scan after right lung lavage was performed; **(C)** HRTC scan after WLL was completed.

The patient was immediately supinated, and bilateral lung ventilation was restored with a rapid increase of SpO_2_ up to 91%. The ABG performed showed severe respiratory acidosis (pH 7.07, pO_2_ 94.2 mmHg, pCO_2_ 114.5 mmHg, HCO_3−_ 25.3 mmol/L); blood pressure was 115/65 mmHg, heart rate 98 bpm. Subsequently, the patient was admitted to the intensive care unit and, after the normalization of gas exchange and resolution of respiratory acidosis, extubated in 36 h. During the second procedure, a complete left lung lavage and a partial right lung lavage were performed (Figure 2). The second procedure was better tolerated, and after 24 h ICU, the patient was extubated. In total, 8 L of saline solution (for the right lung) and 7 L of saline solution (for the left lung) were needed to complete the lavage. Chest HRCT (Figure 3C) performed after the WLL showed a dramatic improvement of the GGO and the appearance of a left pleural effusion, possibly secondary to intraoperative hydro trauma, which rapidly responded to diuretic therapy with furosemide.

The patient was discharged without the need for long-term oxygen, with a SpO_2_ of 97% while breathing room air.

## Discussion

Pulmonary alveolar proteinosis (PAP) is a rare disease characterized by a progressive accumulation of a lipoproteinaceous, eosinophilic, periodic acid-Schiff (PAS) positive material (mostly surfactant phospholipids and lipoproteins) within the alveoli, possibly resulting in respiratory failure (1, 2). PAP syndrome is classified as primary PAP [due to the interruption of granulocyte–macrophage colony-stimulating factor (GM-CSF) signaling], secondary PAP (due to a reduction in the number or function of alveolar macrophages), and congenital PAP (due to the production of dysfunctional surfactant) (1, 3).

Among the secondary forms, there is lysinuric protein intolerance (LPI), a rare inherited metabolic disease characterized by defective cationic amino acid (CAA—lysine, arginine, and omithine) transport (4). In LPI, a mutation within the *SLC7A7* gene, which encodes the y + L transport system, leads to reduced renal clearance and intestinal absorption of CAA. Clinically, LPI is characterized by gastrointestinal symptoms, failure to thrive, renal disease, and osteoporosis, all secondary to hyperammonemia (5). Pulmonary involvement may manifest with a range of interstitial lung disease patterns, including PAP in ~15% of cases (4). The mechanisms underlying PAP pathogenesis in LPI are incompletely understood. The main hypothesis refers to a bone marrow-derived monocyte disorder that leads to the formation of dysfunctional alveolar macrophages which, together with the y + L transport system deficiency, causes alveolar accumulation of PAS-positive material (5).

To date, there are no treatments for PAP secondary to LPI (4).

WLL is the standard therapy for patients with PAP and moderate-to-severe symptoms (6, 7). Therapeutic WLL under general anesthesia is a complex procedure, potentially associated with a wide range of complications, the most frequent being severe hypoxemia, convulsions, pneumothorax, and pleural effusion (8). Our case shows that WLL can paradoxically aggravate the baseline hypoxemia, as a result of a V'A/Q' mismatch. To minimize this risk, we used the lateral decubitus position during the procedure to decrease the pulmonary shunt. However, deterioration of gas exchange can occur, and its management should include a prompt interruption of fluid infusion and quick reestablishment of bilateral ventilation in supine decubitus. Furthermore, a rapid instillation of large volumes of fluid can result in the formation of pleural fluid collections that can be managed with diuretic therapy or thoracentesis.

In our case, PAP was secondary to LPI, which makes the number of therapeutic options limited. Disease course varies, and the prognosis is unpredictable (9).

In PAP, 30–50% of patients require only one lavage, while others require repeated lung lavages at 6- to 12-month intervals. The role of WLL remains less defined, and close clinical and radiological follow-up is needed to assess the long-term efficacy of the procedure (1).

Uncertainties regarding the pathogenesis of the disease have severely limited the etiologic treatment of PAP in LPI. To date, there are no data concerning the role of anti–GM-CSF autoantibodies or bone marrow transplantation in PAP secondary to LPI (4, 5).

To the best of our knowledge, this is the first reported case of a life-threatening severe respiratory failure in PAP secondary to LPI, complicated with a pre-existing right pneumothorax, successfully treated with WLL. The role of WLL remains less defined, and close clinical and radiological follow-up is needed to assess the long-term efficacy of the procedure. In the absence of any other possible approved therapy in PAP due to LPI, it is possible to perform a rescue WLL despite it being performed in these severe respiratory conditions with a difficult anesthesiology management and an existing pneumothorax.

## Data availability statement

The original contributions presented in the study are included in the article/supplementary material, further inquiries can be directed to the corresponding author.

## Ethics statement

Written informed consent was obtained from the participant/patient(s) for the publication of this case report.

## Author contributions

SPe and ML: conception of the manuscript, literature search, and drafting of the manuscript. SO, AD, SPe, AG, LA, and SM: literature search. PS and VP: critical review of the manuscript. CR: conception of the manuscript and literature search. All the authors approved the final version of the manuscript.
